# Engineered extracellular vesicles derived from primary M2 macrophages with anti-inflammatory and neuroprotective properties for the treatment of spinal cord injury

**DOI:** 10.1186/s12951-021-01123-9

**Published:** 2021-11-17

**Authors:** Chuanjie Zhang, Daoyong Li, Hengshuo Hu, Zhe Wang, Jinyu An, Zhanshan Gao, Kaihua Zhang, Xifan Mei, Chao Wu, He Tian

**Affiliations:** 1 Department of Orthopedics, The First Affiliated Hospital of Jinzhou Medical University, No. 2, Section 5, Renmin Street, Jinzhou, 121002 Liaoning China; 2 Key Laboratory of Medical Tissue Engineering of Liaoning Province, No. 40, Songpo Road, Jinzhou, 121002 Liaoning China; 3 Pharmacy School, Jinzhou Medical University, No. 40, Songpo Road, Jinzhou, 121002 Liaoning China; 4 Department of Histology and Embryology, Jinzhou Medical University, No. 40, Songpo Road, Jinzhou, 121002 Liaoning China

**Keywords:** Spinal cord injury, Extracellular vesicles, Curcumin, M2 repolarization, Neuroprotection

## Abstract

**Background:**

Uncontrollable inflammation and nerve cell apoptosis are the most destructive pathological response after spinal cord injury (SCI). So, inflammation suppression combined with neuroprotection is one of the most promising strategies to treat SCI. Engineered extracellular vesicles with anti-inflammatory and neuroprotective properties are promising candidates for implementing these strategies for the treatment of SCI.

**Results:**

By combining nerve growth factor (NGF) and curcumin (Cur), we prepared stable engineered extracellular vesicles of approximately 120 nm from primary M2 macrophages with anti-inflammatory and neuroprotective properties (Cur@EVs^−cl−NGF^). Notably, NGF was coupled with EVs by matrix metalloproteinase 9 (MMP9)-a cleavable linker to release at the injured site accurately. Through targeted experiments, we found that these extracellular vesicles could actively and effectively accumulate at the injured site of SCI mice, which greatly improved the bioavailability of the drugs. Subsequently, Cur@EVs^−cl−NGF^ reached the injured site and could effectively inhibit the uncontrollable inflammatory response to protect the spinal cord from secondary damage; in addition, Cur@EVs^−cl−NGF^ could release NGF into the microenvironment in time to exert a neuroprotective effect against nerve cell damage.

**Conclusions:**

A series of in vivo and in vitro experiments showed that the engineered extracellular vesicles significantly improved the microenvironment after injury and promoted the recovery of motor function after SCI. We provide a new method for inflammation suppression combined with neuroprotective strategies to treat SCI.

**Graphical Abstract:**

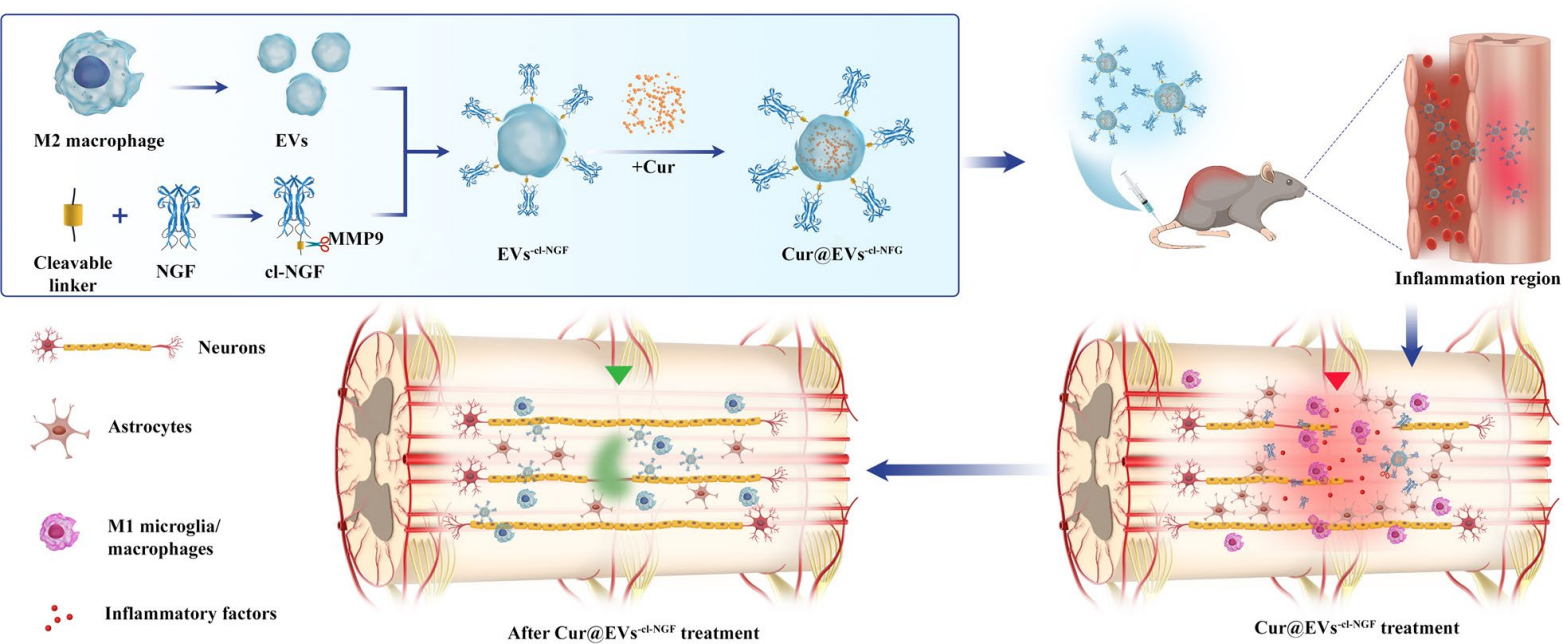

**Supplementary Information:**

The online version contains supplementary material available at 10.1186/s12951-021-01123-9.

## Background

Spinal cord injury (SCI), associated with complicated pathological changes and irreversible nerve damage, is one of the most destructive diseases [1]. Patients often experience a permanent loss of motor and sensory functions, leading to a decreased quality of life and increased economic burden on the family and society [2]. SCI is characterized by a primary injury caused by acute and focal lesions (including the destruction of the vasculature and the blood-spinal cord barrier) and extensive secondary injury composed of ischemia, hypoxia, and uncontrolled neuroinflammatory reactions [3, 4]. Anti-inflammatory and neuroprotective strategies are still the starting point of current SCI drug treatment [5, 6]. Therefore, anti-inflammatory drugs and neurotrophic drugs are usually included in the treatment regimen for SCI [7]. However, due to the short half-life, low solubility, and low accumulation at the injured site, it is difficult for clinical medications to exert good anti-inflammatory and neurotrophic effects [8].

In recent years, extracellular vesicles, as a new type of drug carrier, have attracted widespread attention from researchers [9]. Increasing numbers of studies have shown that extracellular vesicles, with advantages of high biocompatibility, low cytotoxicity, immune inertia, and long-term circulation, have broad application prospects in improving drug delivery and therapeutic effects [10, 11]. M2 macrophages play a crucial inhibitory effect on inflammation in various inflammatory diseases, including SCI [12]. Interestingly, extracellular vesicles derived from M2 macrophages (EVs) can inherit diverse anti-inflammatory cytokines, such as interleukin-10 (IL-10) and transforming growth factor (TGF-β), as well as various chemokine receptors from the macrophage surface, such as chemokine receptor type 2 (CCR2) [13]. More significantly, EVs have played a central role in treating inflammatory diseases, such as atherosclerosis, skin damage and stroke [13, 14]. Thus, EVs with both inflammation suppression and targeting functions are ideal drug carriers. Previous research by our team has confirmed that EVs carrying berberine can effectively reduce the inflammatory response after SCI to promote the recovery of motor function [15].

Nerve growth factors (NGF) can reduce neuronal apoptosis, promote angiogenesis, and promote axon extension and functional recovery [16, 17]. After SCI, hypoxia–ischemia leads to a significant decrease in endogenous expression of NGF at the injury site, highlighting the importance of delivering exogenous NGF [18, 19]. However, previous research has indicated that the biological half-life of NGF after intravenous injection is approximately 2.3 h, so poor bioavailability significantly limits the accumulation of NGF in target cells [20]. To prolong the circulation time of NGF in the body, we coupled NGF to the surface of EVs by the matrix metalloproteinase 9 (MMP9) cleavable linker (cl), which had a sequence of RVGLP, to prepare EVs^−cl−NGF^ [21, 22]. Considering that MMP9 expression is significantly increased at the injury site after SCI, NGF in EVs^−cl−NGF^ can dissociate from EVs and be released into the microenvironment over time [23, 24]. The NGF is then taken up by nerve cells through NGF receptor-mediated endocytosis, avoiding consumption by inflammatory cells [25].

Curcumin (Cur) is a natural polyphenol with anti-inflammatory properties that promote the polarization of M1 macrophages to M2 macrophages [26, 27]. Simultaneously, low solubility, poor stability, low absorption rate, and short biological half-life lead to low bioavailability, limiting its anti-inflammatory application in vivo [28, 29]. To overcome these shortcomings, Cur was loaded into EVs^−cl−NGF^, and Cur@EVs^−cl−NGF^ was prepared to treat SCI.

**Fig. 1:**
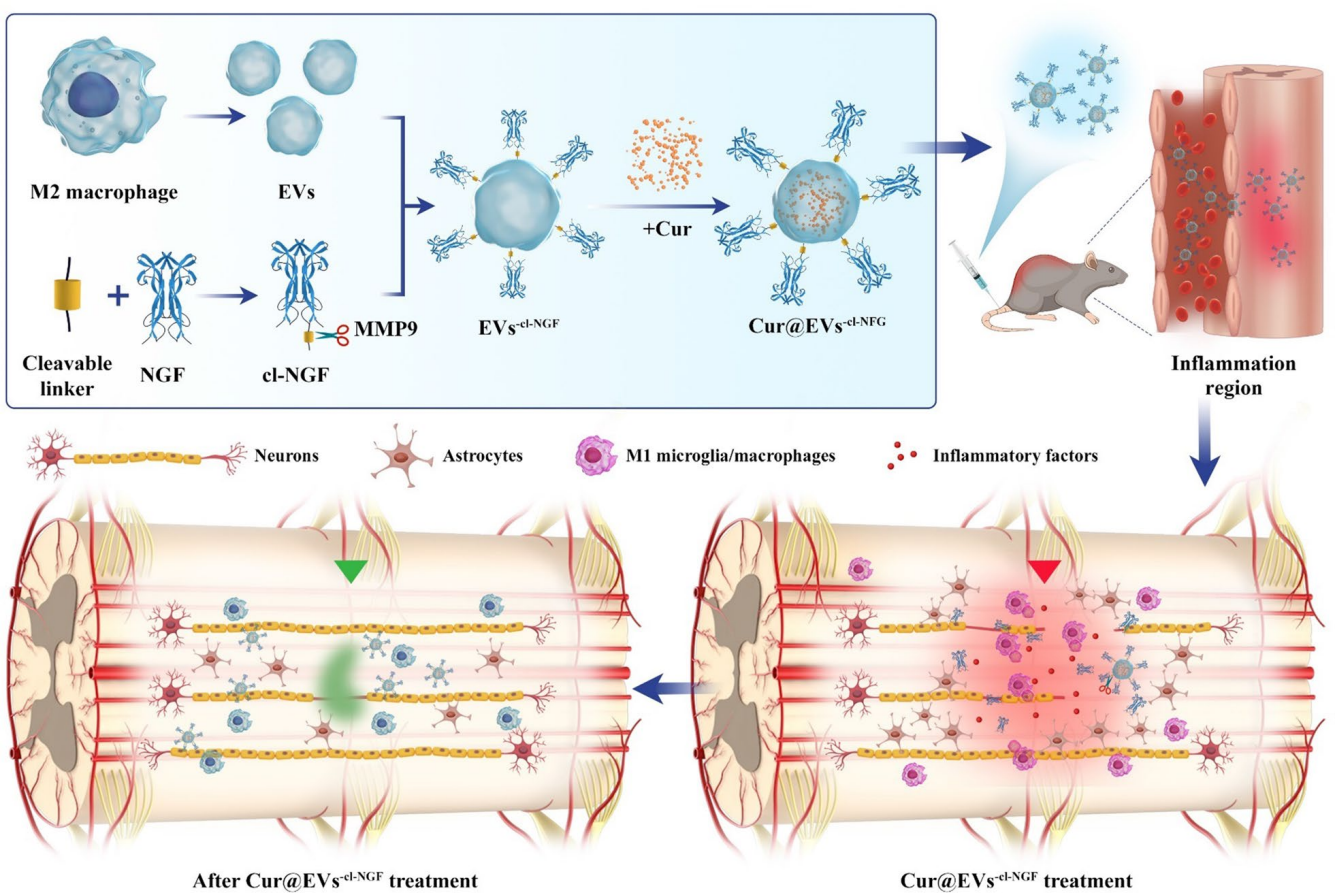
Scheme of engineered extracellular vesicles derived from primary M2 macrophages preparation and spinal cord injury therapy

In this work, the designed Cur@EVs^−cl−NGF^ had both anti-inflammatory and neuroprotective properties. The design concept and mechanism of action are shown in the schematic diagram below (Fig. 1). These effects were verified in vitro using cell experiments and in vivo using animal experiments. We hope that this design can provide new ideas for the clinical treatment of SCI.

## Results

### Preparation and characterization of EVs^−cl−NGF^

**Fig. 2:**
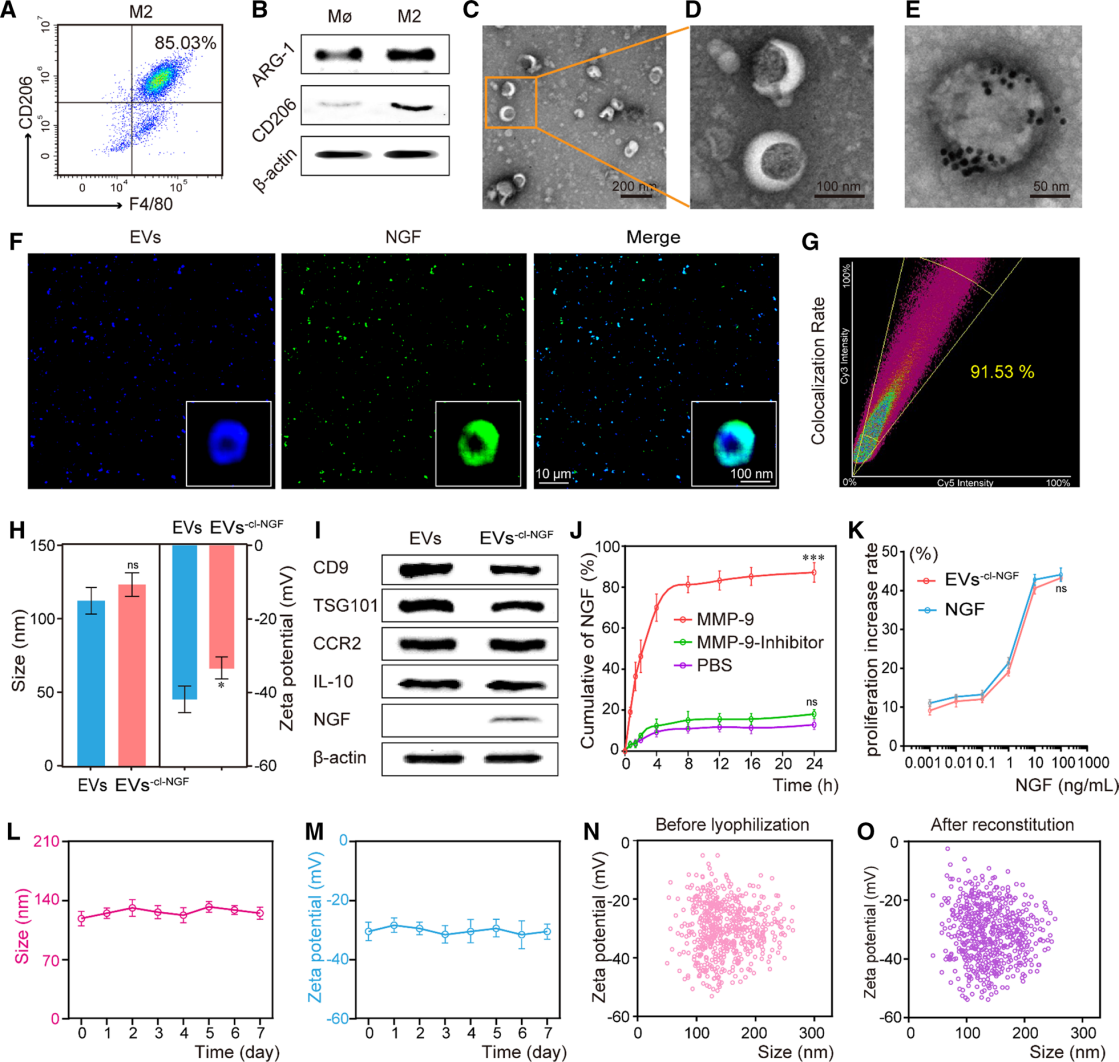
Preparation and characterization of EVs^−cl−NGF^. **A** Flow cytometry analysis of primary macrophages at 48 h after treatments with IL-4. (Numbers indicated per cent F4/80 + , CD206 + cells). **B** Western blotting analysis of primary Mø and M2 macrophages. ARG-1 and CD206 was a marker of M2 macrophages. **C**, **D** TEM imaging of EVs. **E** TEM images of EVs^−cl−NGF^ bound with secondary immune-antibody-linked Au nanoparticles. **F** CLSM images and the STED images (enlarged illustration in the lower right corner) of EVs^−cl−NGF^, EVs (blue) and NGF (green). **G** The colocalization rate of EVs and NGF. **H** The size and zeta potentials of EVs before and after loading NGF. **I** Western blotting analysis of EVs and EVs^−cl−NGF^. CD9 and TSG101 was a marker of extracellular vesicles, IL-10 was a functional protein of M2 macrophages, and CCR2 was Inflammatory chemotactic protein. **J** The release rate of NGF under different conditions (PBS, MMP9 + inhibitor, or MMP9). **K** Comparison of the activity of NGF released from EVs^−cl−NGF^ to the original NGF. **L**, **M** The stability of particle size and zeta potentials of EVs^−cl−NGF^ in PBS for 7 days. **N**, **O** Before lyophilization and after reconstitution, the particle size and Zeta potential of EVs^−cl−NGF^. Data presented the mean ± SD (n = 3 per group) (*P < 0.05, ***P < 0.001 and ns: not significant)

After IL-4 (20 ng/mL) stimulation for 48 h, flow cytometry showed that approximately 85.03% of primary macrophages obtained from the abdominal cavity of mice had differentiated into M2 macrophages with anti-inflammatory effects (Fig. 2A). Next, we used western blotting to detect M2 macrophage marker proteins (ARG-1 and CD206). The results further verified that M2 macrophages were induced successfully (Fig. 2B). Transmission electron microscopy (TEM) showed the classic morphology of EVs with an average diameter of ~ 120 nm (Fig. 2C, D). Then, the successful connection of NGF and EVs through a metalloproteinase 9 (MMP9) cleavable linker was verified by binding secondary antibody-linked Au nanoparticles (Fig. 2E). The high degree of colocalization of NGF and EVs under CLSM and STED further validated the success of the modification (Fig. 2F). The proportion of colocalization was approximately 91.53% (Fig. 2G). Nanoparticle tracking analysis (NTA) showed that after modification with NGF, the size of EVs^−cl−NGF^ was not statistically increased compared with that of EVs, while the absolute value of zeta potential was significantly reduced to 33.56 mV from 42.17 mV (Fig. 2H). Western blotting was used to detect the expression of exosome marker proteins (CD9, CD63, and TSG101) in EVs^−cl−NGF^ and EVs [30]. Negative expression of ApoA1 and Golgi in EVs^−cl−NGF^ proved the absence of contaminating extracellular particles and intracellular material in EVs preps (Additional file 1: Figure S9). Then the expression of chemokine receptors (CCR2) mediating the chemotaxis of exosomes to inflammatory lesions and inflammation inhibitory cytokines (IL-10) in EVs and EVs^−cl−NGF^ were further detected by western blotting. We found that the modified EVs' marker and functional protein expression did not change significantly (Fig. 2I). Based on ELISA kits, we detected the release ability of NGF from EVs^−cl−NGF^. After adding MMP9 to the PBS solution of EVs^−cl−NGF^, the NGF linked on the surface of EVs was effectively dissociated. The solution with MMP9 and the MMP9 inhibitor GM6001 together did not dissociate NGF, which showed that the cleavable linker was specific to MMP9 (Fig. 2J). Subsequently, the PC12 cells growth rate was determined with an MTT assay. Comparing the NGF released from EVs^−cl−NGF^ with the free form of NGF, we found no significant difference between free NGF and NGF in EVs^−cl−NGF^ in promoting the proliferation of PC12 cells. This result indicated that the binding and release of NGF did not damage the activity of NGF (Fig. 2K). Finally, we tested the stability of EVs^−cl−NGF^, and the results showed that the size (Fig. 2L) and zeta potential (Fig. 2M) of EVs^−cl−NGF^ did not change significantly after storage in PBS at 4 °C for 7 d. Moreover, after lyophilization and reconstitution, the size and zeta potential of EVs^−cl−NGF^ changed only slightly (Fig. 2N, O). These results indicated that EVs^−cl−NGF^ had the stability needed for future clinical applications.

### The ability of EVs^−cl−NGF^ to target sites of inflammation and release NGF.

**Fig. 3:**
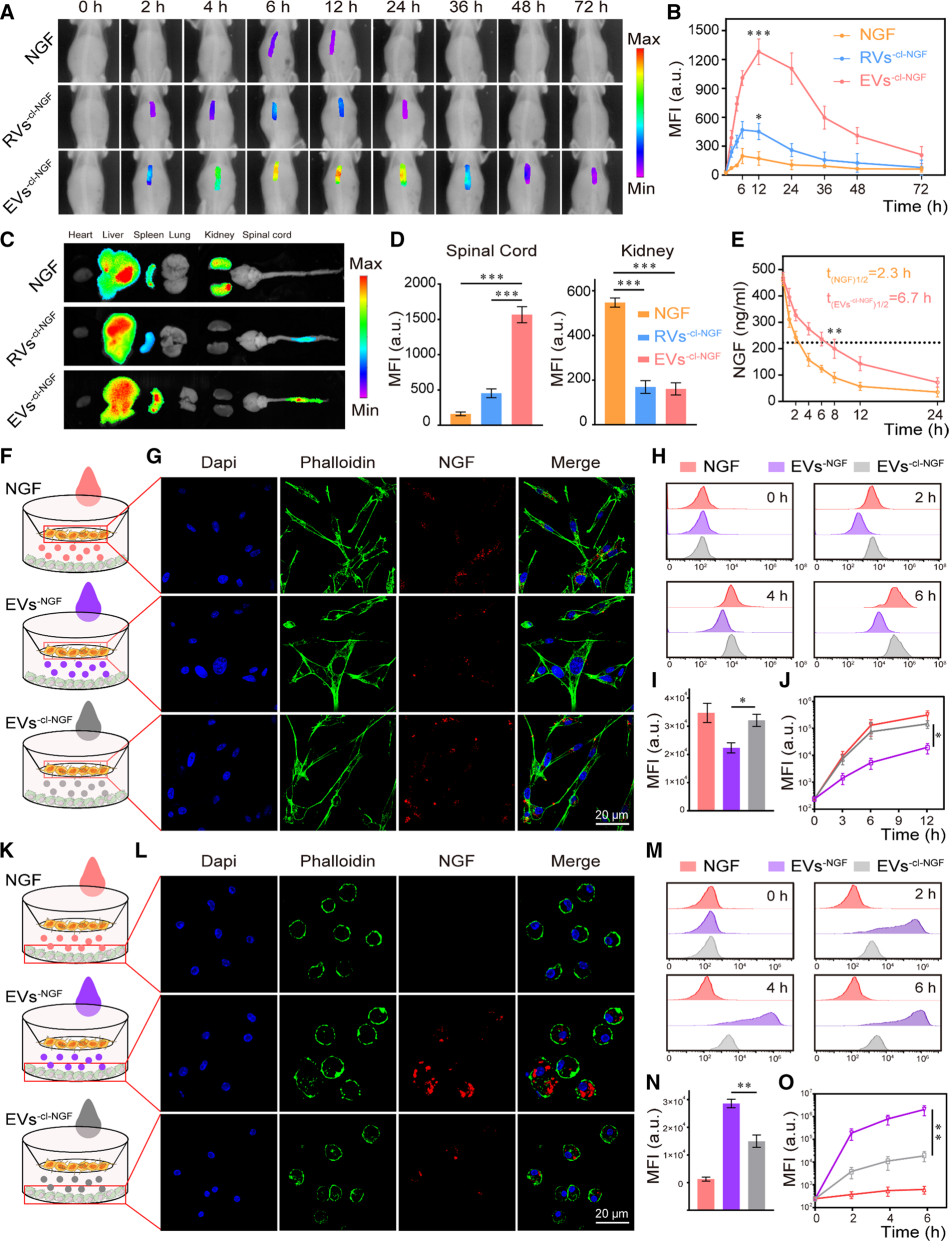
The ability of EVs^−cl−NGF^ to target sites of inflammation and release NGF. **A** NGF fluorescence imaging in the injured spinal cord of different group mice after NGF, EVs^−cl−NGF^ and RVs^−cl−NGF^ injected by tail vein respectively, in different time points. **B** Fluorescence quantitative analysis of NGF inside injured spinal cord in different group mice at different time points. **C** The fluorescence imaging of the vital organs (heart, liver, spleen, lung and kidney) and spinal cord from different groups of mice at 12 h after tail vein injection. **D** Fluorescence quantitative analysis of spinal cord and kidney in different groups. **E** The concentration of NGF in plasma after injection of free NGF and EVs^−cl−NGF^ at different times. **F**, **K** Schematic diagram of the coculture and the uptake of primary macrophages and PC12 cells in different groups. **G** CLSM images of NGF uptake in PC12 cells cultivated at the upper level of the Transwell™ coculture system. **H** Flow cytometry analysis of NGF uptake by PC12 cells cultivated at the upper level of the Transwell™ coculture system. **I** Fluorescence quantitative statistical analysis of Figure **G**. **J** Fluorescence quantitative statistical analysis of Figure **H**. **L** CLSM images of NGF uptake in primary M1 macrophages cultivated at the lower level of the Transwell™ coculture system. **M** Flow cytometry analysis of the uptake of NGF by primary M1 macrophages cultivated at the lower level of the Transwell™ coculture system. **N** Fluorescence quantitative statistical analysis of Figure **L**. **O** Fluorescence quantitative statistical analysis of Figure **M**. Data presented the mean ± SD (n = 6 per group) (*P < 0.05, **P < 0.01, ***P < 0.001 and ns: not significant)

To evaluate whether EVs^−cl−NGF^, as a carrier of NGF, could effectively deliver NGF to the injured site, we used a living imaging system to detect the inflammation targeting ability of EVs^−cl−NGF^ in SCI animal models. In order to better evaluate the active targeting ability of EVs, red blood cell vesicles (RVs) without CCR-2 were used as a negative control (Additional file 1: Fig. S10). After SCI, the mice were randomly divided into 3 groups, NGF, RVs^−cl−NGF^, or EVs^−cl−NGF^ (NGF in each group was labeled with CY7 fluorescent dye), and different nanoparticles were injected through the tail vein. A living imaging system showed that the free form of NGF hardly accumulated at the injury site, and RVs^−cl−NGF^ could exist at the injury site for a shorter time owing to the passive targeting ability of the red blood cell membrane nanoparticles. However, EVs^−cl−NGF^ exhibited the most vigorous fluorescence intensity and the longest accumulation time at the injury site, demonstrating the ability of EVs^−cl−NGF^ to target inflammation (Fig. 3A). Quantitative statistics of the fluorescence image demonstrated that NGF delivered by EVs^−cl−NGF^ accumulated at the injured site within 2 h after injection, reached a peak at approximately 12 h after injection and persisted for more than 72 h (Fig. 3B). Fluorescence images of the main organs (heart, liver, spleen, lung and kidney) and spinal cord from different groups were collected 12 h after tail vein injection, further indicating the satisfactory targeting ability of EVs^−cl−NGF^ for the injured spinal cord (Fig. 3C). Statistical analysis showed that the fluorescence intensity of EVs^−cl−NGF^ was approximately eight times that of free NGF in the spinal cord, while the kidneys of mice injected with free NGF had fluorescence accumulation (Fig. 3D). An NGF ELISA kit was used to detect the biological half-life of NGF in the blood of each group. The results showed that the circulation time in the body of NGF in EVs^−cl−NGF^ was significantly prolonged due to the long-term circulating ability of EVs (Fig. 3E) [15]. Next, we constructed the Transwell™ coculture system to verify whether the cleavable linker contributed to NGF uptake by nerve cells. The upper layer of the Transwell™ coculture system consisted of PC12 cells, and the lower layer was primary M1 macrophages. Then, we added free NGF, EVs^−NGF^, or EVs^−cl−NGF^ (NGF in each group was labeled with FITC) to the Transwell™ coculture system. The schematic diagram is shown (Fig. 3F, K). CLSM images showed that the fluorescence intensity of PC12 cells was significantly lower in the EVs^−NGF^ group than in the groups free NGF and EVs^−cl−NGF^ (Fig. 3G), while the fluorescence intensity of M1 macrophages cultured in the lower layer was significantly higher than that in the groups free NGF and EVs^−cl−NGF^ (Fig. 3L). Fluorescence intensity statistical analysis further demonstrated that compared with the EVs^−NGF^ group, the amount of NGF endocytosis increased significantly in PC12 cells in the EVs^−cl−NGF^ group (Fig. 3I) but was reduced in M1 macrophages (Fig. 3N). This finding suggested that due to the presence of a cleavable linker, NGF of EVs^−cl−NGF^ could be released into the microenvironment to be taken up by PC12 cells in time to reduce the consumption of NGF by M1 macrophages. Subsequently, we used flow cytometry to verify the endocytosis of NGF in different groups over time in PC12 cells or M1 macrophages (Fig. 3H, M). Quantitative analysis of flow cytometry indicated that NGF from EVs^−cl−NGF^ and free NGF had a faster rate of endocytosis and greater uptake by PC12 cells (Fig. 3J), while M1 macrophages showed relatively few uptakes (Fig. 3O).

### In vitro inflammation regulation and neuroprotective capabilities of EVs^−cl−NGF^

**Fig. 4:**
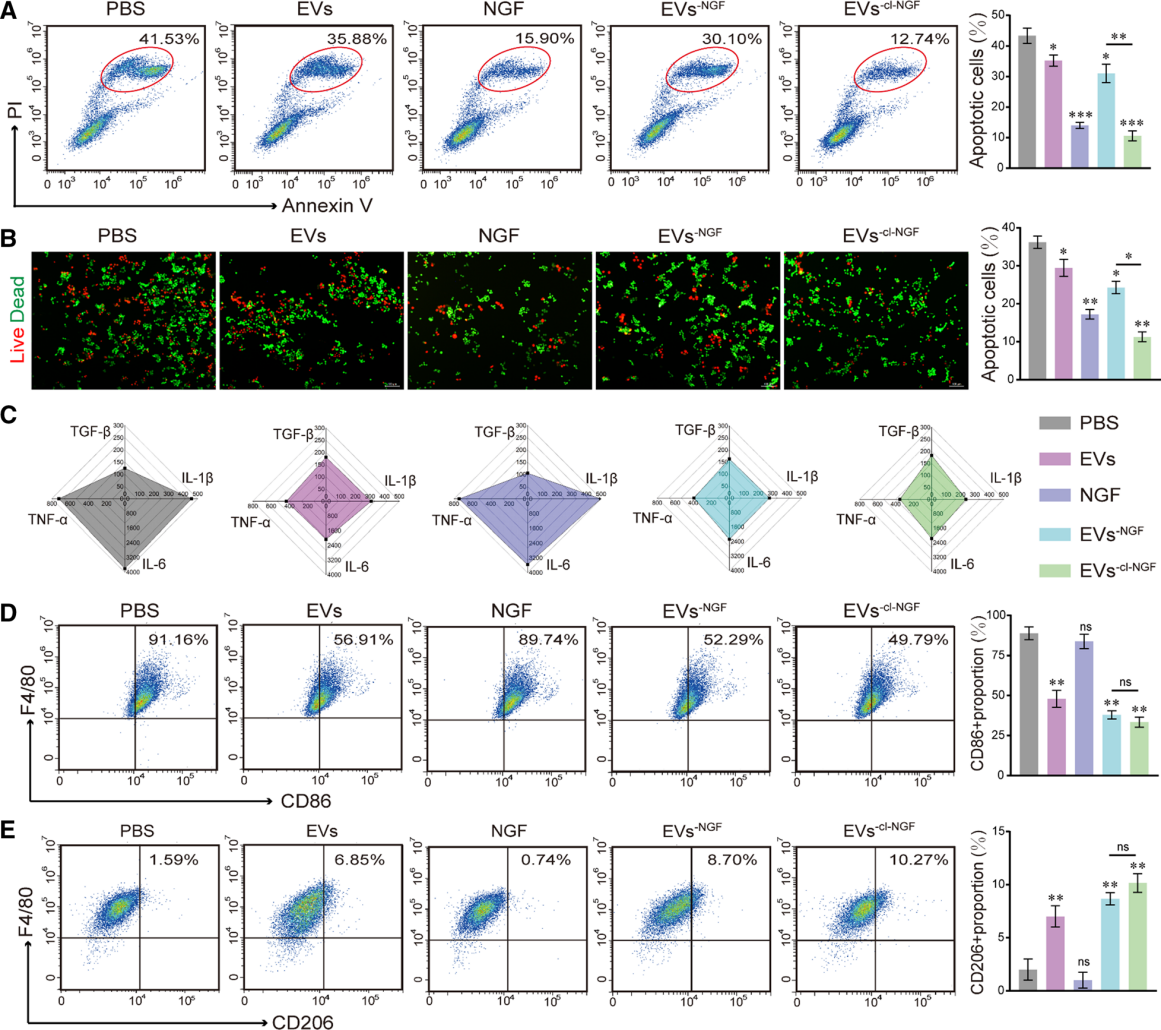
In vitro inflammation regulation and neuroprotective capabilities of EVs^−cl−NGF^. **A** Flow cytometry analysis of the neuroprotection of EVs^−cl−NGF^ on PC12 cells cultivated at the upper level of the Transwell™ coculture system. (Numbers in the areas indicated per cent late apoptotic cells). **B** Live-Dead cell staining to evaluate the neuroprotection of EVs^−cl−NGF^ on PC12 cells cultivated at the upper level of the Transwell™ coculture system. **C** ELISA-based measurement of TGF-β, IL-1β, IL-6, and TNFα (pg/mL) in supernatants from the culture medium from different groups. **D** Flow cytometry analysis of primary M1 macrophages cultivated at the lower level of the Transwell™ coculture system. (Numbers indicated per cent F4/80 + , CD86 + cells). **E** Flow cytometry analysis of primary M2 macrophages cultivated at the lower level of the Transwell™ coculture system. (Numbers indicated per cent F4/80 + , CD206 + cells). * at the top of each group's statistical graph indicates the difference analysis with the PBS group. Data presented the mean ± SD (n = 6 per group) (*P < 0.05, **P < 0.01, ***P < 0.001 and ns: not significant)

To test the anti-inflammatory and neuroprotective capabilities of EVs^−cl−NGF^, we conducted a Transwell™ coculture tests. Briefly, primary macrophages were induced to differentiate into M1 macrophages with LPS for 24 h and then cultured in the lower layer of the system. After H_2_O_2_ pretreatment for 12 h to simulate oxidative stress after SCI, PC12 cells were cultured in the upper layer of the system. After adding different nanoparticles to the system for 12 h, we evaluated the survival rate of PC12 cells and the degree of inflammation inhibition in each group. Flow cytometry (Fig. 4A) and live/dead cell staining (Fig. 4B) were used to detect the survival rate of PC12 cells under various treatment conditions. The flow cytometry results showed that under the action of EVs^−cl−NGF^, the apoptotic rate of PC12 cells decreased from 41.53 to 12.74%. The statistical results of live/dead cell staining further confirmed the neuroprotective effect of EVs^−cl−NGF^. Notably, compared with the PBS group, the survival rate of PC12 cells in the EVs group was also significantly improved, which suggested that inhibiting the inflammatory response in the system might be beneficial to the survival of injured nerve cells. Simultaneously, based on ELISA kits, we detected the expression of proinflammatory factors (TNF-α, IL-1β, and IL-6) and anti-inflammatory factors (TGF-β) in the supernatants (Fig. 4C) (Supplementary data for these graphs is in Additional file 1: Figure S6). As expected, in the EVs, EVs^−NGF^, and EVs^−cl−NGF^ groups under the action of EVs, the expression of proinflammatory factors (TNF-α, IL-1β, and IL-6) was significantly reduced, and the expression of anti-inflammatory factors (TGF-β) was increased. Flow cytometry detection of the macrophage phenotype showed that the proportion of M1 macrophage subpopulations (CD86 +) dropped to 49.79% from 91.16% in the EVs^−cl−NGF^ group, suggesting that LPS induction was significantly blunted (Fig. 4D). Moreover, the proportion of M2 macrophage subpopulations (CD206 +) rose to 10.27% from 1.59% (Fig. 4E). It was rather remarkable that the EVs group and EVs^−NGF^ group also had inflammation inhibitory effects under the action of EVs.

### Cur@EVs^−cl−NGF^ showed better anti-inflammatory effects via Cur delivery

**Fig. 5:**
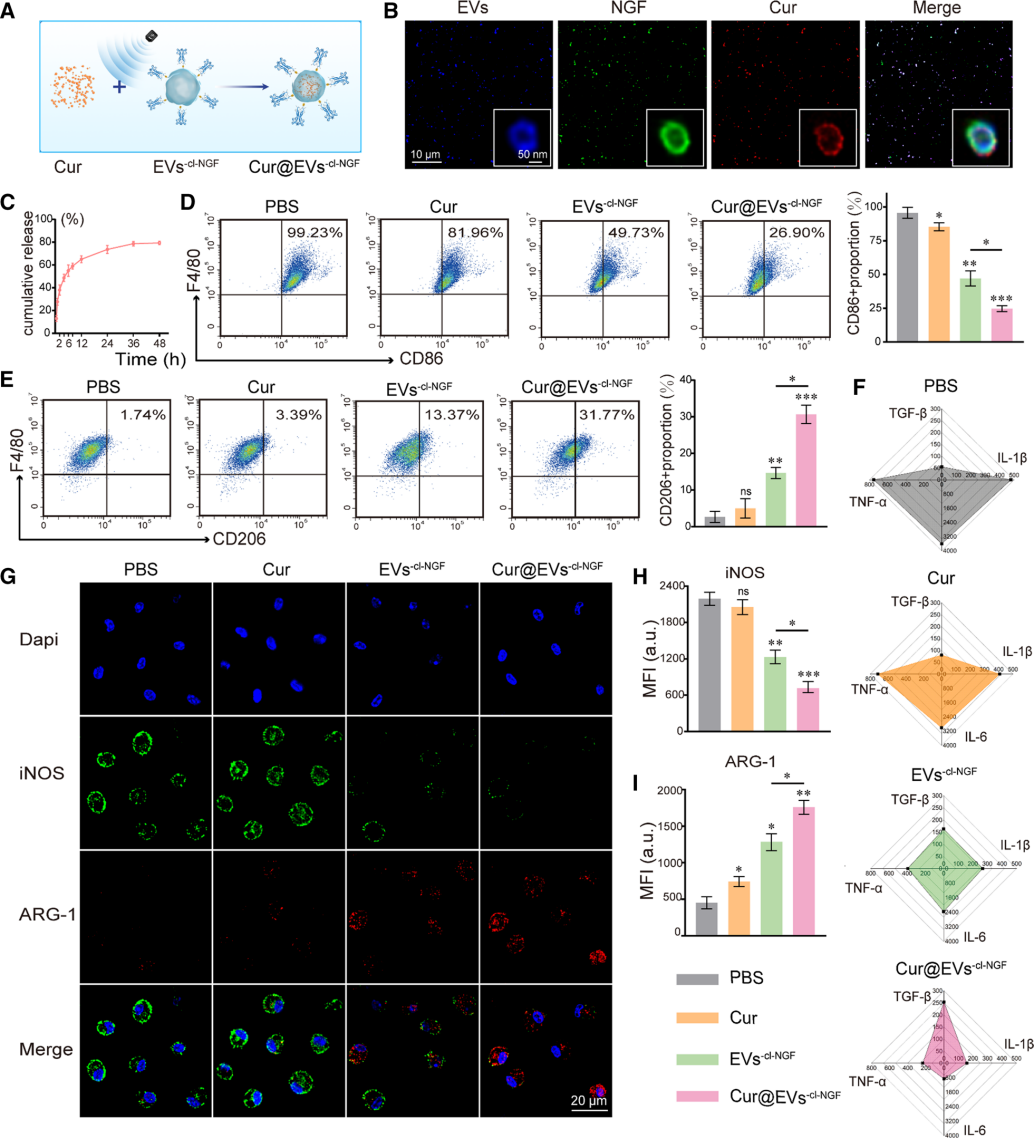
The inflammation regulation capabilities of Cur@EVs^−cl−NGF^. **A** Schematic diagram of Cur loading into EVs^−cl−NGF^. **B** CLSM images and the STED images (enlarged illustration in the lower right corner) of Cur@EVs^−cl−NGF^, EVs (green), NGF (red) and Cur (blue). **C** In vitro Cur release of Cur@EVs^−cl−NGF^. **D** Flow cytometry analysis of primary M1 macrophages cultivated at the lower level of the Transwell™ coculture system. (Numbers indicated per cent F4/80 + , CD86 + cells). **E** Flow cytometry analysis of primary M2 macrophages cultivated at the lower level of the Transwell™ coculture system. (Numbers indicated per cent F4/80 + , CD206 + cells). **F** ELISA-based measurement of TGF-β, IL-1β, IL-6, and TNFα (pg/mL) in supernatants prepared from the culture medium. **G** Representative fluorescence images of the macrophage phenotypes. **H** Fluorescence quantitative statistical analysis of iNOS of Figure **G**. **I** Fluorescence quantitative statistical analysis of ARG-1 of Figure **G**. * at the top of each group's statistical graph indicates the difference analysis with the PBS group. Data presented the mean ± SD (n = 6 per group) (*P < 0.05, **P < 0.01, ***P < 0.001 and *ns* not significant)

As a small molecule drug, although Cur has anti-inflammatory and antioxidant functions, its poor solubility and low bioavailability limit its effectiveness in vivo [27]. Therefore, we opted for Cur as a model drug of small molecule drugs to enhance the anti-inflammatory function of EVs^−cl−NGF^. The process of loading Cur into EVs^−cl−NGF^ by the ultrasonic method is shown in the schematic diagram (Fig. 5A). We calculated that the EN% of Cur is about 37.68%. CLSM and STED were used to detect whether Cur was successfully loaded into EVs^−cl−NGF^. The results showed that NGF, EVs, and Cur presented a high degree of colocalization, indicating the success of the loading (Fig. 5B). Conducting the drug release test in vitro, we found that Cur in Cur@EVs^−cl−NGF^ showed slow release behavior in vitro. At 48 h, the cumulative release reached approximately 78.84% (Fig. 5C). Then, we used flow cytometry to examine the effect of Cur@EVs^−cl−NGF^ on the repolarization of M1 macrophages located in the lower layer of the Transwell™ coculture system. It was rather remarkable that compared with the EVs^−cl−NGF^, the ratio of M1 macrophage subpopulations (CD86 +) was reduced in the Cur@EVs^−cl−NGF^ group to 26.90% (Fig. 5D), and the ratio of M2 macrophage subpopulations (CD206 +) also increased to 31.77% (Fig. 5E). This result suggested that by loading Cur, Cur@EVs^−cl−NGF^ achieved a better anti-inflammatory effect, which would be more suitable for regulating inflammatory diseases than EVs^−cl−NGF^. In addition, ELISA kits were used to detect the levels of proinflammatory factors (TNF-α, IL-1β, and IL-6) and anti-inflammatory factors (TGF-β) in the supernatant. As expected, under the combined effect of Cur and EVs, the Cur@EVs^−cl−NGF^ group showed the best anti-inflammatory effect (Fig. 5F) (Supplementary data for these graphs is in Additional file 1: Figure S6). To more intuitively observe the changes in macrophage polarization-related proteins (iNOS, ARG-1) at the cellular level between the groups, we performed immunofluorescence staining on primary macrophages in the lower layer of the TranswellTM. The CLSM images showed that compared with the PBS group, the expression of marker protein (iNOS) of M1 macrophage subpopulations was significantly reduced in the EVs^−cl−NGF^ group and Cur@EVs^−cl−NGF^ group, and the expression of marker protein (ARG-1) of M2 macrophage subpopulations was significantly increased (Fig. 5G). Subsequently, fluorescence quantitative statistical analysis showed that the expression of iNOS in the Cur@EVs^−cl−NGF^ group was lower than that in the EVs^−cl−NGF^ group (Fig. 5H). Moreover, the expression of ARG-1 showed the opposite result to iNOS, which further confirmed that Cur@EVs^−cl−NGF^ had the most potent regulatory effect on macrophage polarization (Fig. 5I).

### Cur@EVs^−cl−NGF^ promotes the functional recovery of mice with SCI

**Fig. 6:**
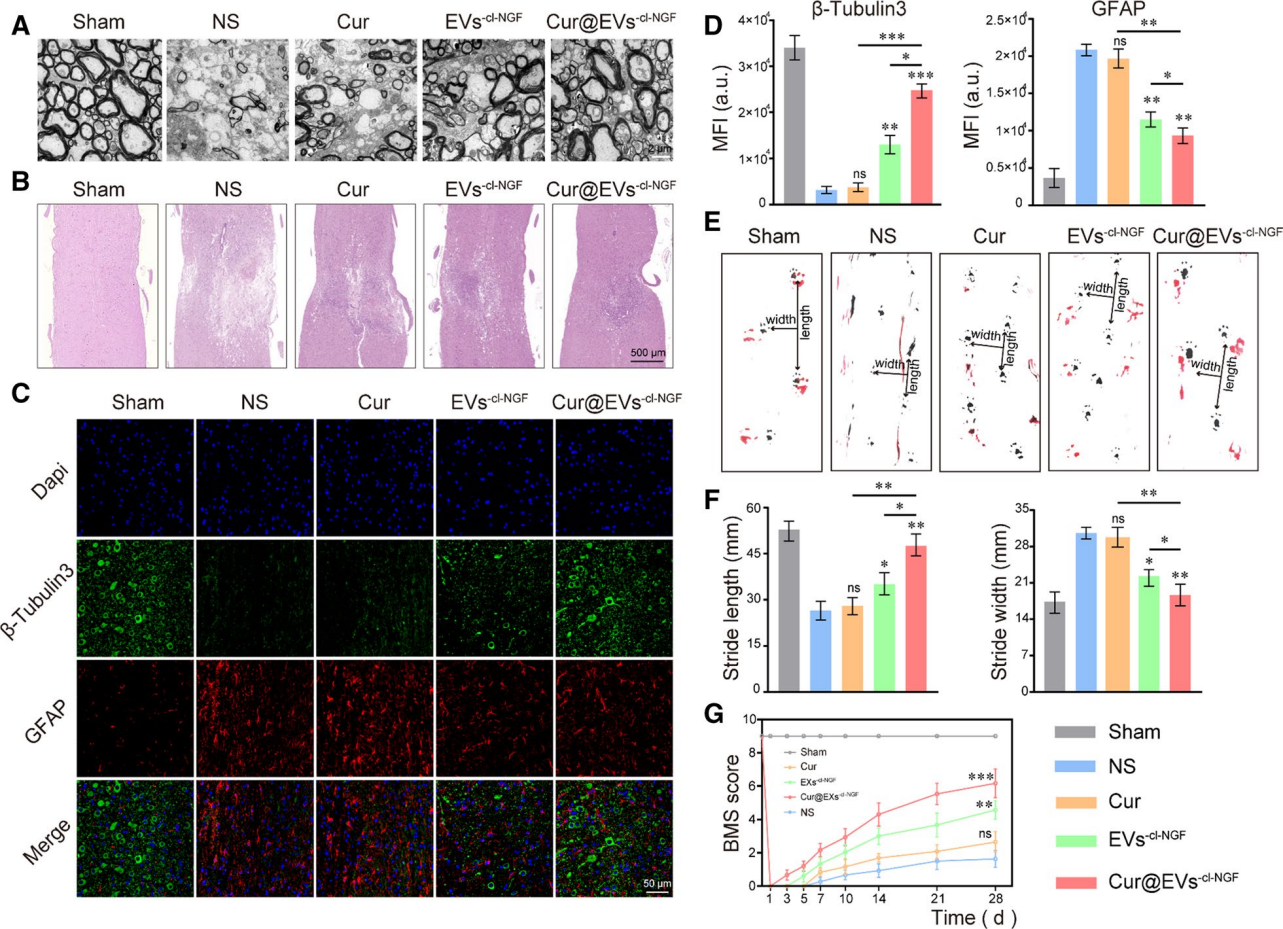
Cur@EVs^−cl−NGF^ promotes the recovery of motor function after SCI. **A** Ultrastructure of myelin sheaths from different groups determined by TEM. **B** H&E stained images of spinal cord tissue from different groups. **C** Fluorescence images of spinal cord tissue, in which the green channel represented neuronal axon tubulin (β3-Tubulin) and the red channel represented astrocytes (GFAP). **D** Fluorescence quantitative statistical analysis of β3-Tubulin and GFAP in figure **C**. **E** Representative images of footprint behavioral assays. **F** Quantitative analysis of stride length and stride width in footprint assays. **G** Mice motor functional recovery of different groups mice during treatment using BMS scores for statistical analysis. (n = 12 per group). * at the top of each group's statistical graph indicates the difference analysis with the NS group. Data presented the mean ± SD (n = 6 per group, except for data **G**) (*P < 0.05, **P < 0.01, ***P < 0.001 and *ns* not significant)

Mice with SCI were randomly divided into 5 groups: (1) Sham, (2) Normal saline (NS), (3) Cur, (4) EVs^−cl−NGF^, and (5) Cur@EVs^−cl−NGF^ to explore the therapeutic effect of Cur@EVs^−cl−NGF^. Since the inflammatory reaction occurs immediately and increases significantly within 3–6 h after the injury [31, 32], we started administering treatment within 2–3 h after SCI. We selected 2 h, 2, 4, and 6 d after the injury as the administration time, and the mice were sacrificed at 28 d after injury to evaluate the recovery status of each group. First, we assessed the ultrastructure of myelin sheaths of mice that received different treatments by TEM (Fig. 6A). The photo showed that the myelin sheaths in the sham group were arranged in a tight and ordered manner, the thickness was uniform, and the layered structure was evident. The ultrastructure of myelin sheaths in the mice treated with NS was the most severely damaged. However, after Cur treatment, the amelioration of myelin sheaths was very limited. The therapeutic effect of free NGF and individual EVs is also unsatisfactory (Additional file 1: Figure S12). After EVs^−cl−NGF^ and Cur@EVs^−cl−NGF^ treatments, the myelin sheaths' numbers, diameters and thickness were significantly improved. Specifically, the protective effect of Cur@EVs^−cl−NGF^ on myelin sheaths was superior. The spinal cord from each group of mice was subjected to H&E staining to investigate further the lesion changes at the injury site (Fig. 6B). The results showed that compared with the NS treatment group, the spinal cord in the Cur@EVs^−cl−NGF^ treatment group was significantly more complete, its lesions were significantly reduced, and pathological changes were significantly improved. Moreover, both the number of surviving neuronal axon tubulin and the severity of scars caused by astrocytes were closely related to the recovery of motor function in injured mice. Therefore, we observed differences in the expression of GFAP and β3-Tubulin in each group of mice at 28 d after injury by immunofluorescence staining (Fig. 6C). The statistical analysis of β3-Tubulin fluorescence showed that the number of surviving microtubule axon proteins in the EVs^−cl−NGF^ and Cur@EVs^−cl−NGF^ groups was significantly higher than that in the NS groups and Cur groups; in particular, the Cur@EVs^−cl−NGF^ group had the most robust expression of β3-Tubulin (Fig. 6D). The fluorescence statistics of GFAP showed that scar formation in the EVs^−cl−NGF^ and Cur@EVs^−cl−NGF^ groups was significantly less than that in the NS and Cur groups (Fig. 6D). These results further confirmed the therapeutic effect of Cur@EVs^−cl−NGF^ on mice with SCI. Finally, we evaluated the effects of different treatments on motor function recovery through behavioral analysis. Footprint behavioral assays showed that the hind footprints were clearly on the ground in the sham group with a steady stride, the forefoot stride length was the longest, and the stride width was the narrowest (Fig. 6E). In contrast, the footprints of the NS group were the poorest. The hind feet showed a dragging gait, the forefoot stride length was significantly shortened, and the stride width was significantly increased. Moreover, the gait of the hindfoot and the stride length and width of the forefoot showed the most improvement in the Cur@EVs^−cl−NGF^ group (Fig. 6F). In addition, the BMS scores at multiple time points after injury in each group of mice further confirmed that the Cur@EVs^−cl−NGF^ group had a therapeutic effect on spinal cord injury (Fig. 6G).

### The role of Cur@EVs^−cl−NGF^ in the early stages of injury

**Fig. 7:**
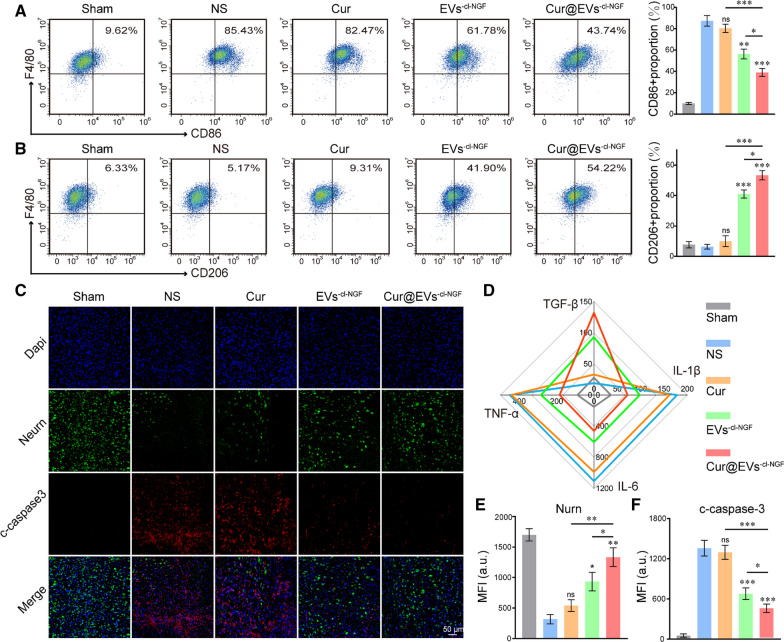
The role of Cur@EVs^−cl−NGF^ in the early stages of injury. **A** Flow cytometry analysis of the ratio of M1 microglia/macrophages to total microglia/macrophages. (Numbers indicated per cent F4/80 + , CD86 + cells). **B** Flow cytometry analysis of the ratio of M2 microglia/macrophages to total microglia/macrophages. (Numbers indicated per cent F4/80 + , CD206 + cells). **C** Fluorescence images of spinal cord tissue, in which the green channel represented neurons (NurN) and the red channel represented apoptosis marker protein (c-caspase3). **D** ELISA-based detection of proinflammatory factors (TNF-α, IL-1β, IL-6) and anti-inflammatory factors (TGF-β) in the injured spinal cord tissue. **E**, **F** Fluorescence quantitative statistical analysis of NurN and c-caspase3 in figure **C**. * at the top of each group's statistical graph indicates the difference analysis with the NS group. Data presented the mean ± SD (n = 6 per group) (*P < 0.05, **P < 0.01, ***P < 0.001 and *ns*: not significant)

We further explored the internal mechanism of Cur@EVs^−cl−NGF^ treatment for SCI. Spinal cord tissues from each group were removed 7 d after injury to observe the pathological changes. The spinal cord 5 mm above and below the injury point was collected to make a single-cell suspension. The flow cytometry results showed a changed polarization state of monocytes/macrophages in the spinal cord tissue. The proportion of M1 monocytes/macrophages (F4/80 + , CD86 +) was significantly higher than M2 monocytes/macrophages (F4/80 + , CD206 +) in the NS group. Among them, the proportion of M1 monocytes/macrophages in total monocytes/macrophages increased to approximately 85.43%, while the proportion of M2 monocytes/macrophages was only 5%. With different treatments, the polarization state of monocytes/macrophages changed accordingly. Cur@EVs^−cl−NGF^ displayed the most regulatory effect on monocyte/macrophage polarization, balancing the ratio of proinflammatory M1 monocytes/macrophages and anti-inflammatory M2 monocytes/macrophages (Fig. 7A, B). Subsequently, we detected the expression of proinflammatory factors (TNF-α, IL-1β, and IL-6) and anti-inflammatory factors (TGF-β) in the injured spinal cord tissue. The results indicated that compared with the NS treatment group, proinflammatory factors in the spinal cord tissue of the Cur@EVs^−cl−NGF^ group were significantly reduced, while anti-inflammatory factors were significantly increased (Fig. 7D) (Supplementary data for these graphs is in Additional file 1: Figure S6). Moreover, we observed the neuron survival status and apoptosis at the injury point by immunofluorescence staining of the surviving neuronal cells (labeled with anti-NurN antibody) and apoptotic protein c-caspase3 (labeled with anti- c-caspase3 antibody) in each group of mice (Fig. 7C). The NurN fluorescence intensity statistical analysis showed that following treatment of EVs^−cl−NGF^ and Cur@EVs^−cl−NGF^, the number of surviving neurons increased significantly, and the number of surviving neurons was most significant in the Cur@EVs^−cl−NGF^ group (Fig. 7E). The fluorescence intensity statistical analysis of Caspase-3 showed that after Cur@EVs^−cl−NGF^ treatment, apoptosis of the lesion was significantly improved (Fig. 7F). In summary, Cur@EVs^−cl−NGF^ suppressed the inflammatory response by balancing the polarization ratio of macrophages and improved the survival rate of damaged nerve cells, thereby improving the microenvironment of the injured site and promoting functional recovery after injury.

## Discussion

An anti-inflammatory combined neuroprotection strategy is one of the most promising options for the treatment of SCI [8, 33]. Activation of microglia is one of the earliest inflammatory reactions after injury, and this activation is usually excessive [34]. Many proinflammatory factors are released into the damaged microenvironment, and many mononuclear macrophages are recruited to the inflammation site to form an uncontrollable inflammatory response [35]. We found that the microglia/macrophages that accumulated at the injury site in the early stage of injury were mainly the M1 type (Additional file 1: Figure S1), which indicated that the polarization of microglia/macrophages was severely imbalanced under the action of proinflammatory factors [36]. Therefore, regulating the polarization direction of microglia/macrophages to achieve the balance between M1 microglia/macrophages and M2 microglia/macrophages is a potential treatment strategy for uncontrollable inflammatory responses in SCI [37]. Previous studies have shown that EVs can change the polarization state of M1-type macrophages after being taken up by M1-type macrophages [15, 38]. We further found that the ratio of monocytes/macrophages in peripheral blood leukocytes increased significantly within 7 d after injury and reached a peak on day 3 (Additional file 1: Figure S2). This finding implies that EVs can actively target inflammation sites and exert inflammatory inhibitory effects [2, 39].

In this study, the first step was to design a stable drug delivery vehicle with anti-inflammatory, neuroprotective, and targeting capabilities. Considering that the selection of the housekeeping protein in extracellular vesicles may be related to the cell-type specific or extracellular vesicles-specific, we referred to other studies on extracellular vesicles derived from M2 macrophages and chosen β-actin as the housekeeping protein to study the expression of characteristic and functional proteins of EVs before and after modification [13, 40]. The results of western blotting and cell proliferation experiments showed that the modification process did not affect the activity of NGF or the function of EVs (Fig. 2I, K). Thus, the anti-inflammatory and targeting functions of EVs will not change with the addition of NGF. In vivo imaging results of the injured site showed that compared with RVs with only passive targeting capabilities, EVs^−cl−NGF^ had a natural tendency to migrate to the injury site (Fig. 3A). The results of fluorescence imaging of the spinal cord and vital organs showed that free NGF was significantly accumulated in the kidneys in addition to the different accumulations in the spinal cord. In contrast, NGF in RVs^−cl−NGF^ or EVs^−cl−NGF^ was heavily accumulated in the spleen, especially in the EVs^−cl−NGF^ group (Fig. 3C). More importantly, apart from its active targeting ability, EVs also significantly prolonged the circulation time in the body of NGF (Fig. 3E). These advantages of EVs^−cl−NGF^ significantly increased the amount of NGF that accumulated at the injured site and significantly improved the bioavailability of NGF. Considering that the target cells of EVs are inflammatory, the timely dissociation of NGF from EVs after reaching the injured site is also significant. It has been reported that after SCI, the concentration of MMP9 at the injured site increases significantly, and we also discovered this phenomenon through ELISA. CLSM and flow cytometry showed that NGF coupled with EVs through a cleavable linker could be effectively taken up by PC12 cells (Fig. 3G, H). Researchers who use EVs as carriers for NGF always overlook the consumption of NGF by inflammatory cells such as microglia/macrophages. Therefore, it is necessary to use cleavable linkers to ensure that NGF reaches the injured site and can be internalized by nerve cells as much as possible instead of macrophages.

In vitro cell experiments confirmed that EVs reduced the expression of proinflammatory factors (TNF-α, IL-1β, IL-6) in the injured microenvironment and increased the secretion of anti-inflammatory factors (TGF-β) (Fig. 4C). Subsequently, we used flow cytometry to analyze the phenotype of the primary macrophages in the Transwell™ coculture system. The results showed that, the number of M1 macrophages was reduced by approximately 40% under the action of EVs, while the number of M2 macrophages was increased by approximately 10%. The results suggested that although EVs significantly inhibited the inflammatory response, the macrophages were still in a proinflammatory polarized state. Thus, using EVs might not be a good to achieve a satisfactory therapeutic effect, so the drug carrier effect of EVs should also be fully utilized. The model drug Cur was loaded into EVs to improve the inflammation-inhibiting effect of the system. As expected, compared with EVs^−cl−NGF^, Cur@EVs^−cl−NGF^ had a more inhibitory effect on proinflammatory M1 macrophages and significantly promoted the secretion of anti-inflammatory factors and the expression of the macrophage marker protein M2 (ARG-1) (Fig. 5F, G). The phenotypic verification results of microglia/macrophages accumulating at the injury site showed that Cur@EVs^−cl−NGF^ rebalanced microglia/macrophage polarization (Fig. 6A). This finding is of great significance for reducing the secondary damage caused by uncontrollable inflammation.

Moreover, NGF coupled with EVs by cleavable linkers could effectively inhibit the apoptosis of PC12 cells after injury. Flow cytometry and CLSM images suggested that although EVs could indirectly inhibit apoptosis of PC12 cells by reducing the inflammatory response [41, 42], the degree of inhibition was minimal (Fig. 4A, B). This result indicated that it was necessary to link NGF on the surface of EVs, which improved the survival rate of nerve cells. The results of in vivo experiments further validated this point. Because of the existence of NGF, Cur@EVs^−cl−NGF^ greatly promoted the survival rate of neuronal cells in the early stage of injury, which increased the possibility of subsequent functional recovery (Fig. 6C). We speculate that the primary mechanism of Cur@EVs-cl-NGF's neuroprotective effect is as follows. First of all, in previous studies, NGF has been proven to have highly effective neuroprotective effects and can effectively promote the recovery of damaged nerve cells. When connected to EV, NGF will be more driven to the injury site. Secondly, the improvement of the inflammatory microenvironment after spinal cord injury will also reduce neuronal cell apoptosis. Specifically, Cur or extracellular vesicles derived from M2 primary macrophages can reduce neuronal cell apoptosis by inhibiting inflammation. Third, in addition to its anti-inflammatory ability, Cur also plays a vital role in repairing damage to the central nervous system. In other words, the Cur released into the microenvironment is equally meaningful for damaged neurons. In summary, Cur@EVs^−cl−NGF^ can increase neuronal cell survival rate through various potential mechanisms, and each mechanism cooperates to produce a therapeutic effect. The results of motor function recovery also illustrated that Cur@EVs^−cl−NGF^ had the best therapeutic effect, although Cur and EVs^−cl−NGF^ also had different degrees of therapeutic effect (Fig. 7). Moreover, Cur@EVs^−cl−NGF^ not only had a therapeutic effect but also presented superior safety (Additional file 1: Figures S4, S5). Combining all the experimental results, we believe that Cur@EVs^−cl−NGF^, with both inflammation targeting and inhibition and neuroprotection, can inspire the clinical treatment of SCI.

## Conclusion

In this study, we designed multifunctional engineered extracellular vesicles (Cur@EVs^−cl−NGF^) to promote the recovery of motor function after SCI. The combination of extracellular vesicles and Cur effectively changed the polarization state of M1 macrophages after injury and limited the damage to spinal cord tissue caused by uncontrollable inflammation. Cur@EVs^−cl−NGF^ also achieved the transfer of exogenous NGF to the injured site and improved the survival rate of injured nerve cells. Both in vivo and in vitro tests demonstrated that the present design could provide new treatment options for SCI.

## Materials and methods

### EVs isolation

Extraction and induction of peritoneal macrophages: The abdominal skin of the mice was disinfected with ethanol, and 1 mL of 5% starch broth solution was injected into the abdominal cavity of each mouse. After 48 h, the mice were sacrificed by cervical dislocation. The peritoneum of the mice was exposed under aseptic conditions, and the peritoneal cavity was injected with 5 mL precooled Dulbecco's Modified Eagle's Medium (DMEM). After gently massaging the mouse's abdomen for 5 min, a 5-mL syringe was used for flushing the peritoneal cavity lavage fluid repeatedly, and then the lavage fluid was recovered. The lavage fluid was centrifuged at 1000 rpm for 10 min, the supernatant was discarded, and the peritoneal cell concentration was adjusted to 1 × 10^6^/mL. One milliliter per well of the cell suspension was inoculated in a 12-well culture plate and placed in an incubator at 37 °C with 5% carbon dioxide and saturated humidity to allow the macrophages to adhere to the wall. After incubating for 2 h to 4 h, the culture supernatant was discarded, and the cells were cultured in DMEM supplemented with exosome-free 10% fetal bovine serum (FBS) and 1% penicillin–streptomycin (PS). (A 0.22 um filter membrane filters FBS, then the filtrate is placed in an ultra-high-speed centrifuge tube. After 4 °C, 120,000 g ultra-high-speed centrifugation for 90 min, the collected supernatant was collected to prepare exosome-free FBS. After the above treatment, the rate of exosome depletion will exceed 95%. (Additional file 1: Figure S13) After 24 h, interleukin-4 (IL-4) (20 μg/mL) was added to the medium to differentiate primary macrophages into M2 macrophages.

EVs isolation: The cell supernatant of primary M2 macrophages was collected and placed in a centrifuge tube, sequentially centrifuged at 300 g for 10 min, 2000 g for 10 min, 10,000 g for 30 min, and 100,000 g for 70 min at 4 °C. The obtained precipitates were suspended in phosphate-buffered saline (PBS) and centrifuged at 100,000 g for 70 min to wash away impurities. The EVs were resuspended in PBS for further characterization.

### The preparation of EVs^−cl−NGF^ and Cur@EVs^−cl−NGF^

Modification of NGF with cleavable peptide substrate (NHS-Arg-Val-Gly-Leu-Pro-(6-Mal), RVGLP-(6-Mal)): The peptide (0.5 mg/mL) was added to the NGF solution (1 mg/mL) at a molar ratio of 50:1, keeping the linker in excess. The mixed solution was incubated at 4 °C in PBS (pH 7.4) overnight. Then, the samples were filtered using a centrifugal filter device (Amicon Ultra-0.5, Millipore Co, Germany) at 7,000 g for 30 min to remove the excess linker. Next, the NGF was linked to EVs. The extracellular vesicles were pretreated with 1 mM TCEP at 37 °C for 30 min to break the disulfide bonds on the surface of the EVs and expose the sulfhydryl groups. Then, “-cl-NGF” was dissolved in PBS (pH 7.4) and reacted with the EVs at 25 °C for 1 h. Finally, the EVs^−cl−NGF^ was washed and collected by dialysis. Cur was dissolved in a 1:1 mixed solution of ethanol and acetonitrile. PBS was added to the mixed solution to a concentration of the organic solvent of 10%. EVs^−cl−NGF^ was added at a concentration of 20% Cur to the solution. Cur@EVs^−cl−NGF^ was prepared by ultrasonic soaking using a 40- kHz and 100-W intermittent ultrasonic cleaner for 15 min and washing by centrifugation at 5000 rpm three times with a 100-kDa ultrafiltration tube. The encapsulation percentage (EN%) of Cur was calculated using the following formula.

$${\text{EN}}\% \, = \, \left( {{1} - {\text{ amount of free Cur}}/{\text{ amount of Cur in Cur}}@{\text{EVs}}^{{ - {\text{cl}} - {\text{NGF}}}} } \right) \, *{1}00\%$$

### Characterization of EVs^−cl−NGF^ and Cur@EVs^−cl−NGF^

Morphology of EVs^−cl−NGF^: The purified extracellular vesicles solution and 4% PFA were mixed 1:1 (total volume 20 μL) and dropped on a clean plastic film to form a droplet, then the front of the electron microscope carbon mesh was buckled on the droplet and left for 20 min. The carbon mesh was washed 3 times with 100 μL PBS for 3 min each time. Subsequently, which was placed in 100 μL of 5% BSA blocking solution and blocked for 10 min. Anti-NGF (Cell Signaling Technology, MA, USA) was diluted with the primary antibody diluent (1:20), and the carbon mesh was placed on a 20 μL primary antibody droplet and incubated for 30 min. Then the colloidal gold-labeled secondary antibody was diluted (1:20) and placed on the carbon mesh for 30 min. After incubation, the secondary antibody was washed with PBS. The carbon mesh was placed on 100ul 1% glutaraldehyde droplets for 2 min and then washed with deionized water 6 times. 10 μL uranyl acetate was negatively stained for the 90 s, and the carbon mesh was dried and tested. Transmission electron microscopy (TEM) images were obtained on a JEM-1400 microscope (JEOL Japan) with an accelerating voltage of 120 kV. The size distribution and zeta potential of EVs/EVs^−cl−NGF^ were analyzed on a Zetaview (Partical Metrix Germany).

Western blot analysis: The expression of special marker proteins of EVs/EVs^−cl−NGF^ was detected using a western blot analyzer with BeyoECL Plus (Beyotime, Beijing, China). The primary antibodies used were as follows: rabbit anti-CD9 (1:1000, Abcam, Cambridge, UK), rabbit anti-TSG101 (1:1000, Abcam, Cambridge, UK), rabbit anti-CD206 (1:1000, Cell Signaling Technology, MA, USA), rabbit anti-IL10 (1:1000, Cell Signaling Technology, MA, USA), rabbit anti-CCR2 (1:1000, Cell Signaling Technology, MA, USA), rabbit anti-ARG-1 (1:1000, Cell Signaling Technology, MA, USA), rabbit anti-NGF (1:1000, Cell Signaling Technology, MA, USA), and mouse anti-β-actin (1:1000, Abcam, Cambridge, UK).

Colocalization analysis of EVs^−cl−NGF^ and Cur@EVs^−cl−NGF^: anti-CD63 antibody and anti-NGF antibody were incubated with EVs^−cl−NGF^ or Cur@EVs^−cl−NGF^ to label EVs and NGF, respectively. EVs^−cl−NGF^ or Cur@EVs^−cl−NGF^ was imaged by confocal laser scanning microscopy (CLSM) (Nikon A1 Japan) and visualized with stimulated emission depletion (STED) (Leica SP8, Germany).

Release and biological activity of NGF from EVs^−cl−NGF^: MMP-9 was added to a PBS solution of EVs^−cl−NGF^ with/without inhibitor, and the supernatant was removed at different time points. After ultrafiltration, the content of NGF in the filtrate was detected with an NGF ELISA kit. The biological activity of NGF was detected by the MTT test in PC12 cells, in which different concentrations of NGF from EVs^−cl−NGF^/free NGF were added to the culture medium of PC12 cells.

Cur release studies: In vitro drug release studies were conducted using PBS (pH = 7.4) as the release agent at 37 °C, to simulate the human body temperature. First, the samples (Cur@EXS^−cl−NGF^) were placed in a dialysis bag, and subsequently, 4 ml of sample was drawn at a predetermined time point, then adding an equal volume to the release medium to continue drug release. The concentration of the drugs in the sample was measured using an ultraviolet spectrophotometer (UV-2000, LANGUAGES, FRANKVILI, WI) at 430 nm, and the cumulative release was calculated.

### Construction of the Transwell™ coculture model

The Transwell™ coculture model was built with Transwell™ plates (pore size of 8 μm, Corning, USA), and 0.5–1 × 10^5^ PC12 cells were loaded in each upper chamber. Primary macrophages were removed from the mouse abdominal cavity and pretreated in the lower chamber with lipopolysaccharide (LPS) (0.1 μg/mL) for 24 h before building the Transwell™ coculture model. All the cells were cultured in DMEM (Gibco, Grand Island, NY, USA) supplemented with fetal bovine serum (10%), penicillin (100 units/mL), and streptomycin (100 μg/mL) (Gibco, Grand Island, NY, USA) at 37 °C in a humidified atmosphere containing CO_2_ (5%).

### ELISA

According to the manufacturer's instructions, ELISA kits determined the concentrations of TNF-α, IL-1β, IL-6, TGF-β, and NGF. In short, standards, samples, antibodies, and HRP-streptavidin were sequentially added to the reaction wells and incubated at 37°C. Finally, the color-developing solution and the stop solution are sequentially added to the reaction wells. The enzyme standard solution determined the OD value, and the concentration of the determined protein was calculated according to the standard curve.

### Apoptosis test

For the apoptosis experiment of PC12 cells, PC12 cells were loaded in each upper chamber and stimulated with 200 μM hydrogen peroxide (H_2_O_2_) for 12 h to simulate damage to cells caused by oxidative stress. After changing to standard medium, primary M1 macrophages were placed in the lower layer of the Transwell™ coculture model for 12 h. Different nanoparticles (PBS, EVs, NGF, EVs^−NGF^, EVs^−cl−NGF^) were added to the different chambers, and after 12 h, the apoptosis rate of PC12 cells and the macrophage polarization level in each chamber were evaluated by flow cytometry and CLSM.

For the Live-Dead cell staining experiment, PC12 cells were collected by digestion centrifugation and prepared into a cell suspension. 100 µl staining working solution (Prepared according to Calcein-AM/PI Live Cell/Dead Cell Double Staining Kit (Solarbio, Beijing, China) instructions) was added to 200 µl cell suspension and incubated at 37 °C for 15 min. Live cells (green fluorescence, 488 nm) and dead cells (red fluorescence, 550 nm) are simultaneously detected under CLSM.

### Flow cytometry

For the cell uptake experiment, a medium containing NGF, EVs^−NGF^, and EVs^−cl−NGF^ (FITC-labeled NGF, 5 μg/mL) was added to the Transwell™ coculture model. At the different time points, cells were removed and resuspended in a standing buffer to assess the fluorescence of different cells in different groups (Beckman Coulter, USA).

For the apoptosis rate of PC12 cells, the Transwell™ coculture model was removed after different stimulations, and the upper PC12 cells were resuspended. A total of 10^5^ PC12 cells were mixed in 100 μL of binding buffer. Cells were stained with Annexin V-Alexa Fluor 488/PI for 15 min at room temperature to assess cell apoptosis.

Macrophage cells were removed for macrophage polarization, and flow cytometry antibodies were added for 30 min at 4 °C. M1 macrophages were labeled with F4/80 (Biolegend, USA) and CD86 (Biolegend, USA), and M2 macrophages were labeled with F4/80 (Biolegend, USA) and CD206 (Biolegend, USA) to evaluate the differentiation of macrophage phenotypes.

### Animals and the SCI model

Adult male C56BL/6 mice (8–10 weeks old, 22–25 g) were used. All animal experiments were approved by the Animal Protection and Use Committee of Jinzhou Medical University. A contusion-induced SCI model was developed by an improved weightlessness method. In brief, after intraperitoneal anesthesia with 1% sodium pentobarbital (50 mg/kg), the mice were fixed on a sterile operating table, fully exposing the T9 spinal cord. Using a 12.5 g impactor device (diameter: 2 mm), the uniform height was 5 cm down to the spinal cord, resulting in a moderate spinal cord contusion. After successful modeling, the surgical incision was sutured layer by layer, and antibiotics were given to prevent infection. From 2 d after surgery, the mouse bladder was massaged twice a day to help them urinate until the mice could urinate spontaneously. Sham-operated mice underwent the same procedures, except for contusion of the spinal cord. To investigate the treatment effect in vivo, 100 μL of 2 mg/mL Cur, EVs^−cl−NGF^, or Cur@EVs^−cl−NGF^ was injected through the tail vein at 2 h, 2, 4, and 6 d after injury.

### In vivo imaging

The preparation of RVs^−cl−NGF^: The fresh blood was added to 0.01 mol/L PBS (pH 7.4), and centrifuged at 3000 r/min at 4 °C for 20 min. Then the supernatant and the white blood cell and platelet layer under the supernatant were discarded. Red blood cells obtained by centrifugation were washed 3 times with precooled PBS (pH 7.4) (4 ℃, 5 000 r/min, 15 min). A hypotonic solution was added to erythrocyte sedimentation and placed at 4 ℃ for 24 h to lyse the erythrocyte. Which was centrifuged at 10,000 r/min for 20 min, then the supernatant was discarded (repeat 3 to 5 times) until there were no visible red blood cells. The precipitate can be added 0.01 mol/L PBS (pH 7.4) to store at − 20 °C. Liposome extruder was used to squeeze the extracted erythrocyte membrane through 10, 5, 1, 0.4, and 0.2 μm extruded membrane in turn repeatedly. The extruded liquid was collected and passed through a dextran gel column to prepare RVs. Then, prepared RVs^−cl−NGF^ according to the method of preparing EVs^−cl−NGF^.

The preparation of NGF labeled in RVs^−cl−NGF^ and EVs^−cl−NGF^: The exact amount of NGF connected to EVs or RVs was incubated with CY7 at 37 °C for 1 h, after which free NGF was removed by Exosomes spin column (MW 3000) (Thermo Fisher Scientific Cat: 4484449) (4 ℃, 650 g/min, 3 min). Then, prepared RVs^−cl−NGF^ and EVs^−cl−NGF^ as described above.

For in vivo imaging of nanoparticles, according to the loading rate of NGF in EVs (12.15%) and RVs (25.23%), NGF (2 mg/mL, 100 μL), RVs^−cl−NGF^ (7.93 mg/mL, 100 μL), and EVs^−cl−NGF^ (16.46 mg/mL, 100 μL) were injected via the tail vein. At different time points, mice were imaged using a Kodak Imaging System FX Pro to evaluate the fluorescence signal distribution in vivo. Twelve hours after tail vein injection, the mice were sacrificed by cervical dislocation, and the mouse heart, liver, spleen, lung, kidney, and spinal cord were removed for fluorescence imaging.

### Immunofluorescence staining

For the cell uptake experiment, a medium containing NGF, EVs^−NGF^, and EVs^−cl−NGF^ (FITC-labeled NGF, 5 μg/mL) was added to the Transwell™ coculture model for 6 h. Cells were removed from the incubator, and the cells were fixed with immune tissue fixative. Finally, the cell membrane was labeled with phalloidin 561, and the cell nucleus was labeled with 4',6-diamidino-2-phenylindole (DAPI).

For immunofluorescent dual-labeling staining, cells or frozen sections were fixed with immune tissue fixative and incubated with Triton X-100 (0.3%) followed by goat serum (10%) for 2 h. The cells were incubated with the first antibody overnight at 4 °C, washed, and then incubated with Alexa Fluor 488 goat anti-rabbit IgG or Alexa Fluor 594 goat anti-mouse IgG (A-11034/A-11005, 1:250, Thermo Fisher Science) for 2 h at room temperature. Next, the cell nucleus was stained with DAPI. The primary antibodies were as follows: rabbit anti-F4/80 (1:200, Abcam, Cambridge, UK), rabbit anti-cleaved-Caspase3 (c-caspase3) (1:1000, Abcam, Cambridge, UK), mouse anti-ARG-1 (1:1000, Cell Signaling Technology, MA, USA), rabbit anti-iNOS (1:1000, Cell Signaling Technology, MA, USA), rabbit anti-NurN (1:50, Cell Signaling Technology, MA, USA), rabbit anti-GFAP (1:200, Cell Signaling Technology, MA, USA), and rabbit anti-β3-Tubulin (1:200, Cell Signaling Technology, MA, USA).

### Behavioral analysis

The Basso mouse scale (BMS) exercise rating scale was used to evaluate the functional recovery of injured animals on days 1, 3, 5, 7, 14, 21, and 28 according to scores ranging from 0 to 9 (9 for complete normality and 0 for complete paralysis). The animals were placed in the open field and observed for 4 min. Three examiners who did not know the mouse grouping observed and scored the mouse's ankle joints, the touch degree of the sole and dorsum of the foot, trunk stability and, tail position. The experiment was repeated three times.

### Statistical analysis

All data are expressed as the average value of the data distribution evaluated by the Shapiro–Wilk test. The Mann–Whitney U test was used for two-group comparisons. One-way ANOVA and Bonferroni post hoc tests were used to compare more than two groups. All data were graphed and statistically analyzed using GraphPad Prism 9. P < 0.05 was indicated with “*”, P < 0.01 was indicated with “**”, and P < 0.001 was indicated with “***”.

## Supplementary Information

**Additional file 1**. Supporting information.

## Data Availability

The datasets used and analysed during the current study are available from the corresponding author on reasonable request.

## Abbreviations

SCI: Spinal cord injury
EVs: Extracellular vesicles derived from M2 macrophages
NGF: Nerve growth factor
Cur: Curcumin
IL-10: Interleukin-10
TGF-β: Transforming growth factor
TNF-α: Tumor necrosis factor-α
IL-1β: Interleukin-1β
IL-6: Interleukin-6
CCR2: Chemokine receptor type 2
MMP9: Matrix metalloproteinase 9
IL-4: Interleukin-4
TEM: Transmission electron microscopy
CLSM: Confocal laser scanning microscopy
STED: Stimulated emission depletion
BMS: Basso mouse scale
