# Supplementary material for: Engineered extracellular vesicles derived from primary M2 macrophages with anti-inflammatory and neuroprotective properties for the treatment of spinal cord injury
Source: J Nanobiotechnology. 2021 Nov 17;19:373. doi: 10.1186/s12951-021-01123-9 (PMC8600922; doi:10.1186/s12951-021-01123-9)
Supplement: Supplementary file 1 — Additional file 1. Supporting information. [file 12951_2021_1123_MOESM1_ESM.docx]

**Engineered extracellular vesicles derived from primary M2 macrophages with** **anti-inflammatory and neuroprotective properties for the treatment of spinal cord injury.**

Chuanjie Zhang, Daoyong Li, Hengshuo Hu, Zhe Wang, Jinyu An, Zhanshan Gao, Kaihua Zhang, Xifan Mei, Chao Wu and He Tian

**
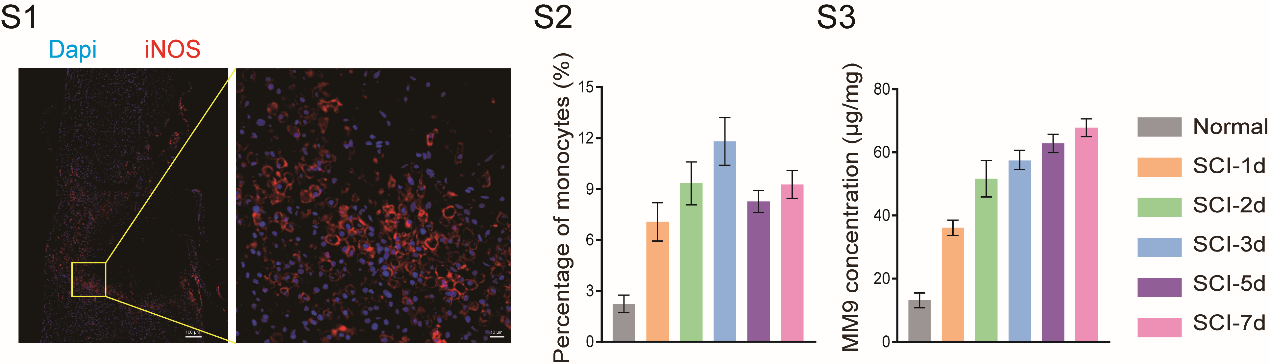
Figure. Supplementary material.** (S1) M1 type macrophages infiltrating the injured site after SCI. (S2) Flow cytometry analysis of the proportion of macrophages to white blood cells in peripheral blood at different times after injury. (S3) Based on ELISA kit, detection of MMP9 concentration changes at damage points after SCI.

**
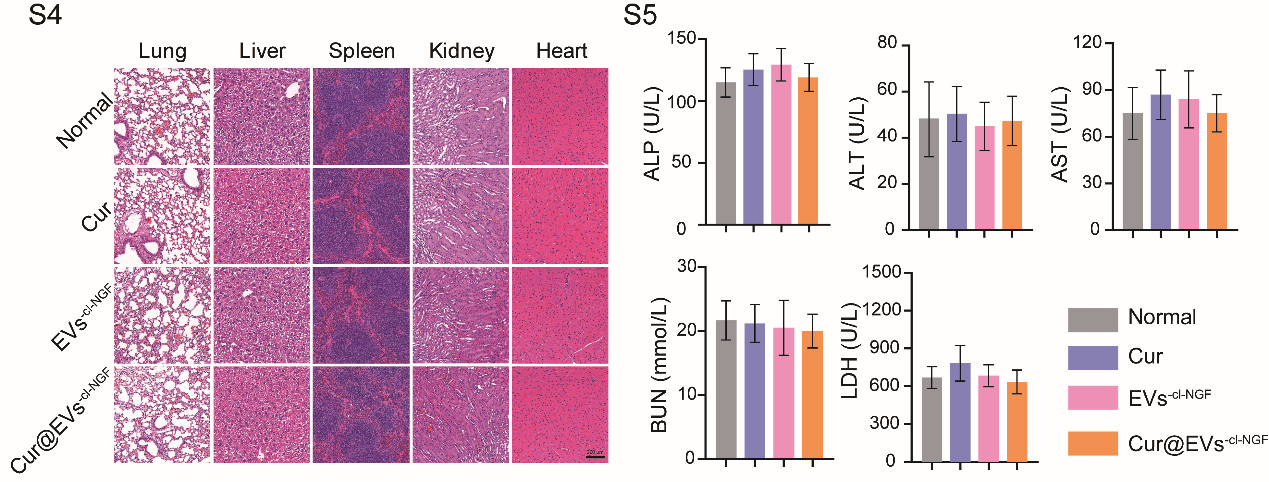
Figure. Supplementary material.** (S4) HE staining of important organs in mice for safety evaluation. (S5) Blood biochemical examination in mice for safety evaluation.


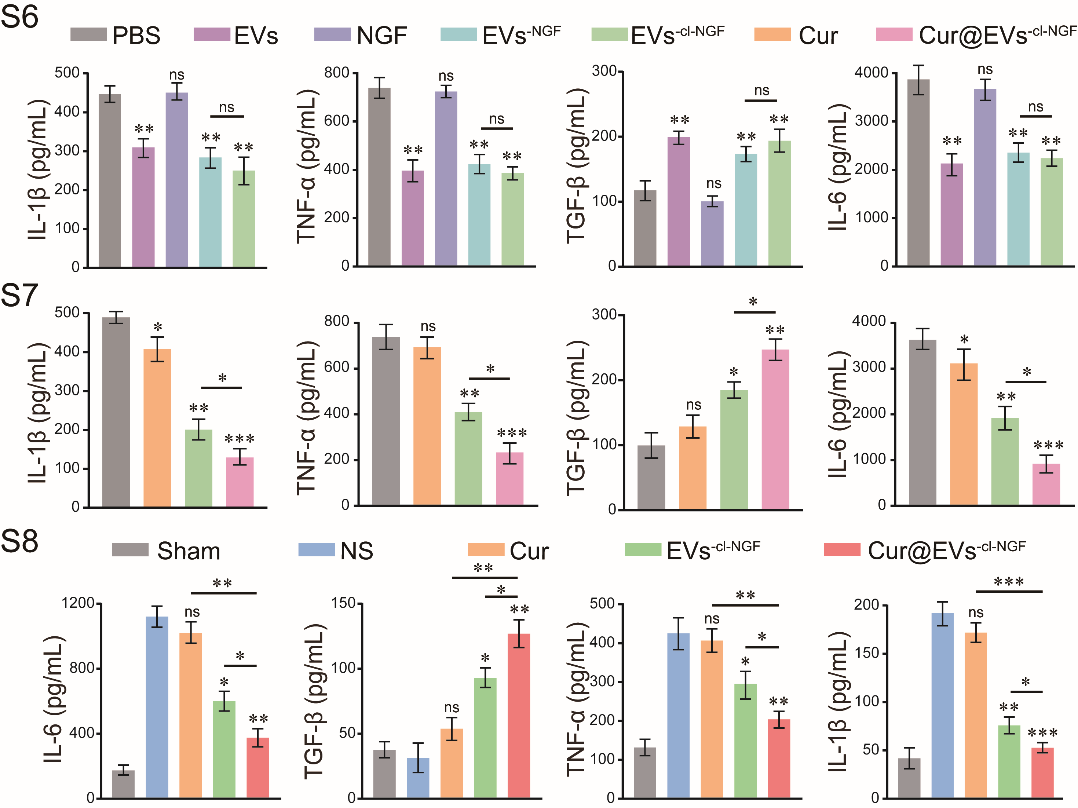


**Figure. Supplementary material.** (S6) Supplementary data for Fig. 4C. (S7) Supplementary data for Fig. 5F. (S8) Supplementary data for Fig. 7D.


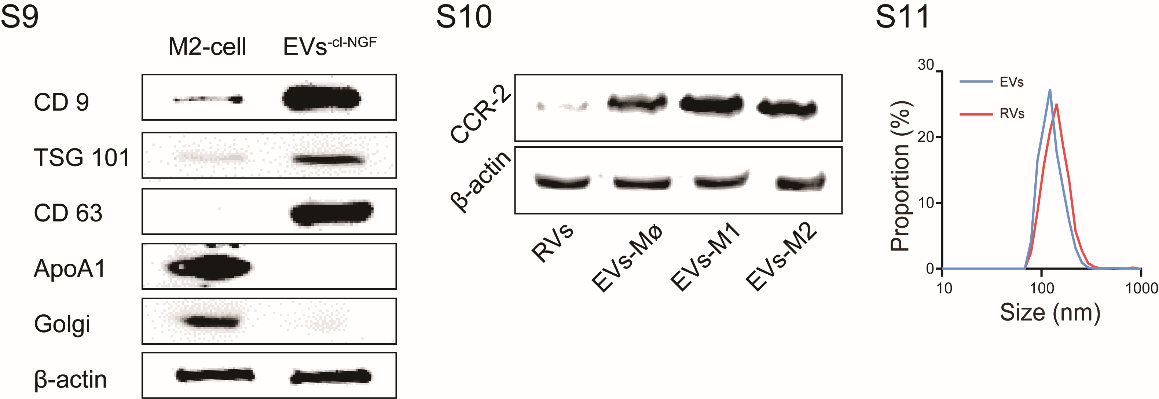


**Figure. Supplementary material.** (S9) Western blotting analysis of primary M2 macrophages and EVs^-cl-NGF^. (S10) Western blotting analysis of the expression of CCR-2 in RVs and EVs derived from primary Mø, M1 and M2 macrophages. (S11) The size comparison of RVs and EVs.


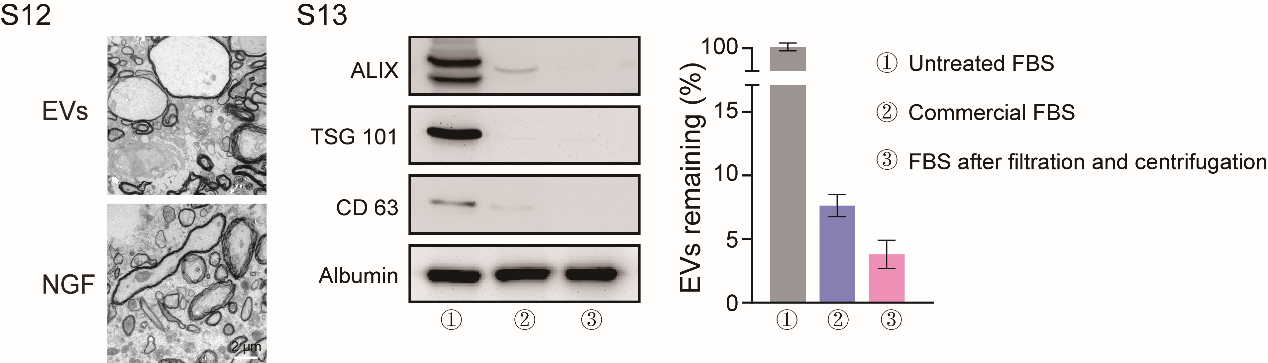


**Figure. Supplementary material.** (S12) Ultrastructure of myelin sheaths determined by TEM. (S13) The comparison of EVs content in FBS before and after filtration and centrifugation and commercial exosome-free FBS (Gibco, A2720801).
